# Metabolism-Associated Hepatotoxicity of Gatifloxacin in Zebrafish Larvae

**DOI:** 10.3390/biom16060780

**Published:** 2026-05-26

**Authors:** Rong Shen, Yichang Yu, Yue Ma, Ran Yu, Rong Lan, Yuning Zhang

**Affiliations:** 1 School of Bioengineering, Beijing Polytechnic University, Beijing 100176, China; mayue@bpi.edu.cn (Y.M.); yuran@bpi.edu.cn (R.Y.); lanrong@bpi.edu.cn (R.L.); zhangyuning@bpi.edu.cn (Y.Z.); 2 Chongqing Research Academy of Eco-Environmental Science, Chongqing 401336, China; yif1123@126.com

**Keywords:** gatifloxacin, zebrafish larvae, hepatotoxicity, lipid metabolism, cytochrome P450, non-oxidative mechanism

## Abstract

Gatifloxacin (GTFX), a fourth-generation fluoroquinolone, causes metabolic disturbances in mammals, but its hepatotoxic mechanisms in aquatic vertebrates remain unclear. This study investigated whether GTFX induces liver injury in zebrafish larvae through oxidative stress or alternative pathways. Larvae at 3 days post-fertilization were exposed to 0.2–2.3 mg/mL GTFX for 48 h. Liver morphology, histopathology, intracellular reactive oxygen species (ROS), and expression of lipid metabolism (*pparg*) and xenobiotic biotransformation genes (*cyp1a*, *cyp1b1*) were assessed. GTFX exposure caused concentration-dependent reductions in liver area, increased hepatic opacity, delayed yolk sac absorption, and hepatocyte swelling with cytoplasmic vacuolization. Despite these structural changes, ROS levels did not differ significantly from those of controls. In contrast, transcriptional analysis revealed significant upregulation of *pparg*, *cyp1a*, and *cyp1b1*, indicating disrupted lipid homeostasis and enhanced detoxification responses. Acute high-dose GTFX exposure induced a metabolism-associated hepatotoxic response in zebrafish larvae, which occurred without a statistically significant change in bulk ROS levels. Together, these findings offer mechanistic insight into fluoroquinolone-associated liver injury.

## 1. Introduction

Fluoroquinolone antibiotics (FQs) are widely used for the treatment of severe bacterial infections [1]. In addition to their well-recognized antibacterial efficacy, a growing body of clinical evidence has associated fluoroquinolone use with systemic adverse effects [2]. Among these adverse effects, hepatic injury has attracted increasing attention, and cases of liver injury associated with fluoroquinolone use have been reported in humans [3]. As a fourth-generation fluoroquinolone, gatifloxacin (GTFX) has been associated with dysglycemia and pancreatic and hepatic disturbances in mammalian systems [4].

Due to their extensive use, chemical stability, and persistence in conventional wastewater treatment, fluoroquinolones are frequently detected as environmental contaminants in the aquatic environment [5,6,7]. Beyond this ecological concern, fluoroquinolones also serve as valuable pharmacological tools for investigating the mechanisms of xenobiotic-induced organ toxicity under controlled laboratory settings. In this context, the zebrafish (*Danio rerio*) has emerged as a well-established vertebrate model for toxicological research. Its utility stems from high genetic homology with humans, rapid organ development, and optical transparency. These features also make it suitable for integrated morphological and molecular analyses [8,9]. Of particular relevance to this study, the zebrafish liver develops early and possesses functional xenobiotic metabolic pathways, making this model especially suitable for investigating the hepatotoxic effects associated with fluoroquinolone exposure [10].

Previous studies have shown that several fluoroquinolones can interfere with cardiovascular, reproductive, and neurological functions in zebrafish and other aquatic organisms [11,12,13]. In comparison, much less is known about how GTFX affects liver structure and function in vivo. Hepatotoxicity caused by xenobiotics is commonly associated with oxidative stress [14]. However, this mechanism alone does not fully account for all forms of drug-induced liver injury, including that associated with certain fluoroquinolones.

Increasing evidence indicates that some pharmaceuticals can disrupt hepatic homeostasis through alternative processes, such as cytochrome P450-related metabolic adaptation and nuclear receptor-mediated regulatory responses [15,16]. Recent integrative reviews emphasize that chemically induced hepatotoxicity frequently involves disruption of hepatic lipid metabolism and regulatory pathways, rather than being driven solely by oxidative stress [17]. These changes may occur independently of, or in parallel with, measurable oxidative stress, pointing to the involvement of non-classical molecular pathways in hepatic toxicity.

Among the regulatory systems involved in hepatic homeostasis, peroxisome proliferator-activated receptor γ (PPARγ) plays a key role in lipid metabolism, energy balance, and inflammatory signaling [18]. Alterations in PPARγ-related pathways have been closely linked to lipid accumulation and metabolic stress in the liver [19,20]. Cytochrome P450 enzymes, particularly members of the CYP1 family, serve as core components of the hepatic xenobiotic biotransformation machinery and are frequently induced as part of an adaptive response to chemical exposure [21,22]. Notably, crosstalk between these pathways has been suggested: alterations in both PPARγ signaling and CYP1-associated metabolic activity are increasingly recognized as integrative markers of metabolic liver perturbation, potentially occurring even in the absence of overt oxidative damage.

Our previous work established that high-dose exposure to GTFX induces cardiovascular toxicity in zebrafish [23]. Building on these findings, the present study was designed to investigate whether GTFX also disrupts hepatic development and function in vivo and to elucidate the underlying molecular mechanisms. By integrating morphological, histopathological, and oxidative stress assessments with gene expression profiling, we sought to test the hypothesis that GTFX-induced hepatotoxicity is primarily driven by disruptions in lipid metabolism and xenobiotic metabolic adaptation, rather than by canonical oxidative stress pathways.

## 2. Materials and Methods

### 2.1. Apparatus and Materials

Gatifloxacin (GTFX, CAS No. 112811-59-3), absolute ethanol, 4% paraformaldehyde, diethyl pyrocarbonate-treated water, hematoxylin, eosin, and dimethyl benzene were purchased from Aladdin Holdings Group (Shanghai, China). Methylcellulose was purchased from Sigma-Aldrich (St. Louis, MO, USA). Chloromethyl-2′,7′-dichlorodihydrofluorescein diacetate (CM-H2DCFDA) was obtained from Life Technologies Corporation (Carlsbad, CA, USA). A FastQuant RT Kit (with gDNase; catalog no. KR106, TIANGEN, China) was used for cDNA synthesis, and an RNA-Quick Purification Kit (catalog no. RN001, YiShan Biotech, Shanghai, China) was used for RNA extraction.

### 2.2. Zebrafish Husbandry

Zebrafish larvae were cultured at and provided by Hangzhou Hunter Biotechnology Inc. Zebrafish (*Danio rerio*) were maintained under standard laboratory conditions in accordance with OECD Test Guideline 236 for fish embryo toxicity testing [24]. Water quality parameters were controlled as follows: temperature 28.0 ± 0.5 °C, pH 6.9–7.2, conductivity 480–510 μS/cm, and hardness 53.7–71.6 mg/L CaCO_3_. Normally developed wild-type AB strain larvae at 3 days post-fertilization (dpf), corresponding to early larval stages beyond embryogenesis, were used for all experiments. All animal procedures were approved by the Institutional Animal Care and Use Committee (IACUC) of the corresponding institution.

### 2.3. GTFX Exposure and Determination of Toxicity Thresholds

A gatifloxacin (GTFX) stock solution (5.0 mg/mL) was freshly prepared each day of use. The compound was dissolved in fish water adjusted to pH 3.7 with hydrochloric acid. The stock was immediately diluted with the same pH-adjusted medium to the desired working concentrations. All dilutions were used within the same day.

The same pH-adjusted medium (pH 3.7) was used for the vehicle control and all treatment groups to minimize pH-related confounding and ensure uniform exposure conditions. This experimental approach followed a previously established zebrafish acute toxicity protocol for GTFX, in which short-term exposure to the pH-adjusted vehicle control alone produced no detectable mortality or overt developmental abnormalities [23].

Zebrafish larvae (3 dpf) were randomly distributed into six-well plates at a density of 30 larvae per well in 3 mL of solution. Six concentrations of GTFX (0.5, 1.0, 2.0, 3.0, 4.0, and 5.0 mg/mL) were tested to determine the lethality threshold. After 48 h of exposure, larval mortality was recorded, and concentration–mortality curves were generated using Origin 8.0 software. The maximum non-lethal concentration (MNLC) and the 10% lethal concentration (LC_10_) were calculated to be 1.7 mg/mL and 2.3 mg/mL, respectively. Although these concentrations exceed typical environmental levels (ng/L–µg/L), they are consistent with acute toxicity testing paradigms for fluoroquinolones in zebrafish [23] and are intended to establish a mechanistic framework under controlled laboratory conditions rather than to mimic environmental exposure.

### 2.4. Assessment of Hepatotoxicity

Based on the lethality thresholds determined above, four concentrations—1/9 MNLC (0.2 mg/mL), 1/3 MNLC (0.6 mg/mL), MNLC (1.7 mg/mL), and LC_10_ (2.3 mg/mL)—were selected to evaluate the hepatotoxic effects of GTFX. These concentrations represent sublethal to near-lethal exposure levels.

For each concentration, 30 zebrafish larvae (3 dpf) were randomly placed into six-well plates, with each well containing 3 mL of exposure medium. A control group treated with pH-adjusted fish water was set up in parallel. After 48 h of exposure, larvae were collected for morphological assessment, histopathological analysis, oxidative stress measurement, and gene expression analysis, as described below.

Given that fluoroquinolone antibiotics are susceptible to photochemical degradation in aqueous media, precautions were taken to minimize light exposure during the experiment. All stock and working solutions were freshly prepared and handled under low-light laboratory conditions. During exposure, culture plates were wrapped in aluminum foil, and larval handling, medium replacement, and sampling were performed under reduced lighting.

### 2.5. Morphological Assessment of Hepatotoxicity

After 48 h of exposure, zebrafish larvae (30 per well) were sampled from each treatment group, and 10 individuals were randomly selected for morphological assessment. Images were captured using a stereomicroscope (Nikon SMZ18, Tokyo, Japan). Liver area, liver opacity, and yolk sac absorption were quantified to assess hepatotoxicity, following established methods for zebrafish hepatotoxicity studies [25]. Prior to imaging, larvae were immobilized in 3% methylcellulose at room temperature for 15 min. Image selection and quantitative analysis (liver area, opacity, and yolk sac area) were performed by an independent researcher blinded to sample grouping, minimizing subjective bias.

### 2.6. Histopathological Analysis

Histopathological analysis was conducted for the control group and the LC_10_ group (2.3 mg/mL). The LC_10_ group was selected for detailed histological evaluation to capture representative hepatic changes at near-lethal exposure levels. Larvae were fixed in 4% paraformaldehyde, dehydrated through a graded ethanol series, embedded in paraffin, sectioned, and stained with hematoxylin and eosin. Liver sections were examined under a light microscope to assess hepatic architecture and cellular morphology.

### 2.7. Oxidative Stress Assay

For the oxidative stress assay, 150 wild-type zebrafish larvae (3 dpf) were randomly divided into five groups (30 larvae each) and exposed to GTFX concentrations of 0.0, 0.2, 0.6, 1.7, and 2.3 mg/mL for 48 h in six-well plates. Following exposure, larvae were incubated with 10 μM CM-H_2_DCFDA at 28 °C for 1 h in the dark to assess intracellular reactive oxygen species (ROS) levels, following a described protocol [26]. CM-H_2_DCFDA, a non-fluorescent probe, diffuses into cells and is deacetylated by intracellular esterases to form 2′,7′-dichlorodihydrofluorescein (DCFH), which is subsequently oxidized by ROS to generate the fluorescent product 2′,7′-dichlorofluorescein (DCF).

After incubation, fluorescence intensity was measured using a Mithras LB940 multifunctional microplate reader (Berthold Technologies, Stuttgart, Germany) at excitation and emission wavelengths of 485 nm and 535 nm, respectively. For each treatment group, ten larvae were randomly selected for measurement. ROS levels were expressed as fluorescence intensity and normalized to the control values to calculate relative changes using the following equation:

(1) $$Relative\;change\;in\;ROS\left(\%\right)=\left(\frac{F_{Sample}{-F}_{Control}}{F_{Control}}\right)\times 100$$

where F_Sample_ represents fluorescence intensity of treated larvae and F_Control_ represents that of the control group.

### 2.8. Gene Expression Analysis

To investigate the molecular mechanisms underlying GTFX-induced hepatotoxicity, transcriptional responses of genes involved in lipid metabolism and xenobiotic biotransformation were analyzed by quantitative real-time PCR (qPCR).

For this analysis, 450 zebrafish larvae at 3 dpf were randomly assigned to six-well plates, with 30 individuals per well in 3 mL of exposure medium. Five experimental groups were established: a control group maintained in fish-rearing water and four groups exposed to GTFX at concentrations of 0.2, 0.6, 1.7, and 2.3 mg/mL. Three independent wells were prepared for each group, serving as three biological replicates.

Following 48 h of exposure, larvae from each well were collected and pooled for RNA extraction, with 30 larvae per biological replicate to ensure sufficient RNA yield and to minimize inter-individual variability. Total RNA was extracted using an RNA Rapid Extraction Kit (catalog no. RN001, YiShan Biotech, Shanghai, China) according to the manufacturer’s instructions. RNA concentration and purity were assessed using a NanoDrop 2000 spectrophotometer (Thermo Fisher Scientific, Waltham, MA, USA).

For each sample, 2 μg of total RNA was reverse-transcribed into cDNA using a FastQuant RT Kit with gDNase (catalog no. KR106, TIANGEN, Shanghai, China). Quantitative PCR was performed using gene-specific primers targeting *actb* (*β-actin*), peroxisome proliferator-activated receptor gamma (*pparg*), cytochrome P450 1A1 (*cyp1a*), and cytochrome P450 1B1 (*cyp1b1*), following a previously described protocol [27]. Primer sequences are provided in Table 1.

Relative gene expression levels were calculated using the 2^−ΔΔCt^ method. *actb* was selected as the reference gene based on its documented stability in the literature [28]. This stability was further confirmed by the comparable Cq values of *actb* across all treatment groups. A melting curve analysis was performed at the end of each qPCR run to confirm the specificity of amplification products.

### 2.9. Euthanasia

At the end of all experiments, zebrafish larvae were euthanized by rapid chilling (immersion in ice-water at 2–4 °C) until death was confirmed, in accordance with accepted euthanasia guidelines for zebrafish embryos and larvae [29].

### 2.10. Statistical Analysis

Data were analyzed using SPSS 26.0 (IBM SPSS Statistics, Chicago, IL, USA) and expressed as mean ± standard error (SE). Normality was assessed using the Shapiro–Wilk test, and homogeneity of variances was evaluated by Levene’s test. For data meeting both assumptions, one-way analysis of variance (ANOVA) was applied, followed by Dunnett’s post hoc test for comparisons between each treatment group and the control. When homogeneity of variances was violated, Welch’s ANOVA with Games–Howell post hoc test was used instead. Student’s t-test was employed for two-group comparisons where applicable. Statistical significance was set at *p* < 0.05. Graphs were constructed using GraphPad Prism 9.0 (GraphPad Software, San Diego, CA, USA).

## 3. Results

### 3.1. MNLC and LC_10_

**Figure 1:**
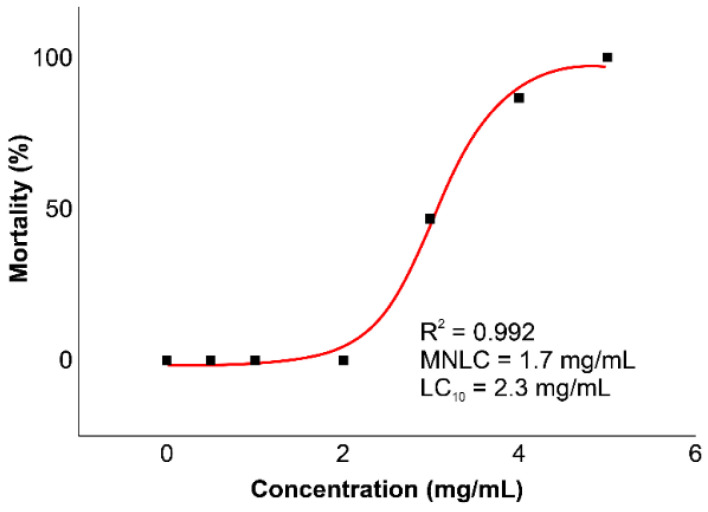
Effect of gatifloxacin (GTFX) on zebrafish mortality.

Larval mortality increased with GTFX concentrations. No mortality was observed at concentrations ≤ 2.0 mg/mL. At 3.0 mg/mL, mortality reached 46.7%, while at 5.0 mg/mL, complete lethality was observed. Based on the concentration–mortality curve, the maximum non-lethal concentration (MNLC) and the 10% lethal concentration (LC_10_) were determined to be 1.7 mg/mL and 2.3 mg/mL, respectively (Figure 1). These concentrations served as reference points for selecting exposure levels in subsequent hepatotoxicity analyses.

### 3.2. Morphological Effects on the Liver

**Figure 2:**
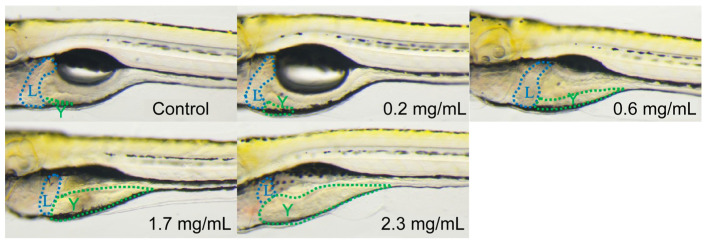
Alterations in the phenotypic features of zebrafish exposed to different concentrations of GTFX. L, Liver; Y, Yolk sac.

After 48 h of exposure, larvae in the control group exhibited a transparent liver with well-defined boundaries and a normal appearance (Figure 2). In larvae exposed to GTFX, morphological changes in the liver were observed in a concentration-dependent manner. At 0.2 mg/mL, the liver remained transparent but with a reduced area compared to the control group. At higher concentrations, the liver area further decreased, the liver tissue appeared darker, and the boundaries of the liver region became progressively less distinct (Figure 2).

**Figure 3:**
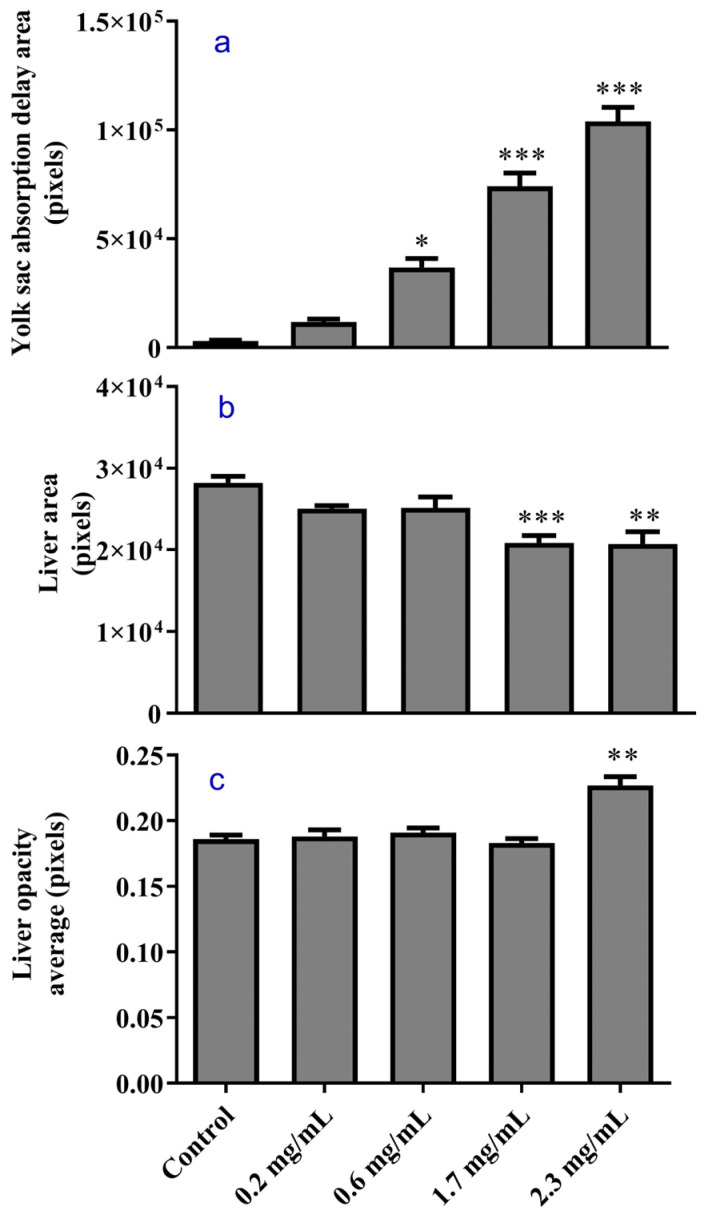
Comparison of (**a**) yolk sac absorption area, (**b**) liver area, and (**c**) liver opacity between the control and GTFX-treated groups. *n* = 10. * *p* < 0.05, ** *p* < 0.01, *** *p* < 0.001 vs. control group.

Quantitative analysis was consistent with these observations. Exposure to 0.6 mg/mL GTFX resulted in a significant increase in yolk sac retention compared with the control group (*p* < 0.05) (Figure 3a). At the MNLC (1.7 mg/mL), yolk sac retention was more pronounced, and liver area was significantly reduced (*p* < 0.001) (Figure 3a,b). At the LC_10_ (2.3 mg/mL), the decrease in liver area was only slightly greater than that observed at the MNLC (1.7 mg/mL). In contrast, the mean liver opacity was significantly increased compared to the other experimental groups (*p* < 0.01). The yolk sac area differed significantly from control values (*p* < 0.001) (Figure 3a–c). A small proportion of mortality (3.3%) was observed in larvae exposed to 2.3 mg/mL GTFX, consistent with its classification as the LC_10_.

### 3.3. Histopathological Changes

**Figure 4:**
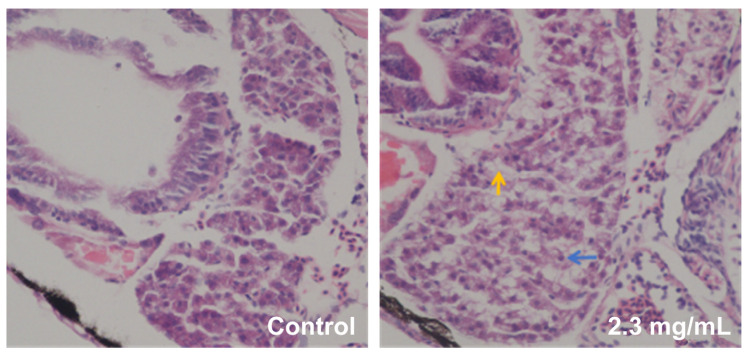
Histopathological examination of zebrafish liver after GTFX exposure. Magnification: 400×. HE staining. The yellow arrow indicates the vacuoles, and the blue arrow demarcates the swelling of the hepatocyte cells.

Histological sections from control larvae exhibited well-preserved hepatic architecture, with hepatocytes displaying uniform cytoplasm and intact cellular morphology. In the LC_10_ group (2.3 mg/mL), hepatocyte swelling and evident cytoplasmic vacuolization were observed (Figure 4). These features indicate structural changes in liver tissue following GTFX exposure at the LC_10_.

### 3.4. Oxidative Stress

**Figure 5:**
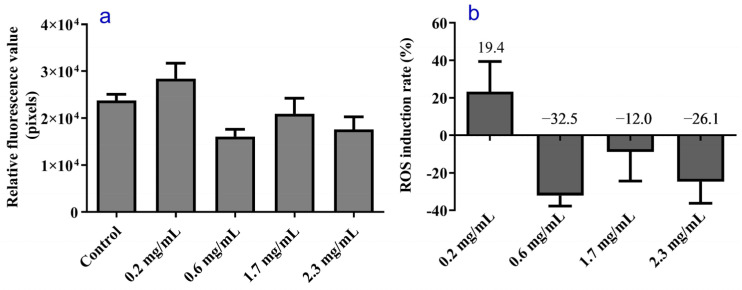
(**a**): Relative fluorescence values (Arbitrary Units, A.U.) in each GTFX-treated group and the control group in zebrafish. Data are expressed as means ± SE. (**b**): Percentage change in ROS levels in zebrafish for each GTFX-treated group compared to the control group. *n* = 30, *p* > 0.05.

Intracellular ROS levels were measured using CM-H_2_DCFDA after 48 h of exposure. Mean fluorescence intensities were 23,826 A.U. in the control group, and 28,449, 16,085, 20,962, and 17,617 A.U. in larvae exposed to 0.2, 0.6, 1.7, and 2.3 mg/mL GTFX, respectively (Figure 5a). Corresponding relative changes in fluorescence intensity were +19.4%, −32.5%, −12.0%, and −26.1% (Figure 5b). None of these differences reached statistical significance compared with the control group (*p* > 0.05). Thus, no statistically significant difference in ROS levels was detected between any exposed group and the control under the present experimental conditions.

### 3.5. Gene Expression

**Figure 6:**
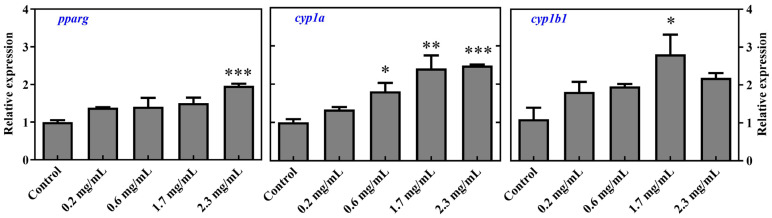
Relative expression levels of *pparg*, *cyp1a*, and *cyp1b1* mRNA. Data are expressed as means ± SE. *n* = 3, * *p* < 0.05, ** *p* < 0.01, *** *p* < 0.001.

Changes in the expression of *pparg*, *cyp1a*, and *cyp1b1* were evaluated after 48 h of GTFX exposure (Figure 6). Relative to the control group (set to 1.00), *cyp1b1* expression levels were 1.82, 1.96, 2.81, and 2.19 in larvae exposed to 0.2, 0.6, 1.7, and 2.3 mg/mL GTFX, respectively. A significant increase in *cyp1b1* expression was observed at 1.7 mg/mL (*p* < 0.05). Expression levels of *pparg* and *cyp1a* also increased following GTFX exposure. For both genes, expression was significantly elevated at 2.3 mg/mL compared with the control group (*p* < 0.001).

## 4. Discussion

In this study, morphological, histopathological, and transcriptional analyses were combined to characterize the hepatic effects of GTFX exposure in zebrafish larvae. The results consistently indicate liver injury accompanied by altered metabolic features, while no significant increase in intracellular reactive oxygen species was detected. Together, these findings suggest that the hepatotoxic effects of GTFX in this model are not primarily driven by oxidative stress but are more closely associated with metabolic disturbance and xenobiotic response pathways.

### 4.1. Effects on Liver Morphology

Morphological alterations of the liver are widely used as early indicators of hepatotoxicity in zebrafish, particularly during larval development. Consistent with this, GTFX exposure in the present study caused a clear, concentration-dependent reduction in liver area, along with increased liver opacity and delayed yolk sac utilization. Similar phenotypic features have been reported in zebrafish models exposed to various hepatotoxic compounds and are generally considered indicative of impaired hepatic development and function [30,31].

Liver area decreased progressively with increasing GTFX concentration. This feature is commonly interpreted as a sign of hepatocellular stress and impaired hepatic growth under xenobiotic challenge [31]. In parallel, the increased liver opacity in GTFX-exposed larvae likely reflects changes in intracellular composition and tissue density. Such changes have previously been linked to lipid accumulation and metabolic imbalance in chemically induced liver injury models [32].

The yolk sac serves as the primary lipid reservoir during early zebrafish development, and efficient hepatic lipid mobilization is essential for normal yolk absorption. Delayed yolk sac utilization, as observed in the present study, therefore suggests impaired hepatic lipid handling and altered metabolic regulation [33,34]. Notably, this phenotype occurred without significant ROS elevation, indicating that metabolic stress, rather than oxidative damage, may underlie the observed developmental disturbances. Taken together, these morphological features support the notion that GTFX interferes with liver metabolic function during early development.

### 4.2. Histopathological Alterations

Histological examination provided further evidence of liver tissue alterations following GTFX exposure. In control larvae, hepatocytes displayed intact cellular architecture with uniform cytoplasm and well-defined boundaries. By contrast, larvae exposed to GTFX at the LC_10_ concentration showed evident hepatocyte swelling and pronounced cytoplasmic vacuolization.

Such histopathological features are commonly observed in livers under metabolic stress and have been associated with lipid dysregulation and disturbed intracellular homeostasis, rather than considered specific markers of primary oxidative injury [35,36]. Similar multi-level structural and molecular alterations have been summarized in recent reviews of drug-induced liver injury, highlighting that hepatocellular damage often arises from metabolic and regulatory disturbances beyond oxidative mechanisms [37]. In the present study, the observed hepatocyte swelling and vacuolization are therefore consistent with a metabolically driven pattern of hepatocellular injury following GTFX exposure.

### 4.3. Oxidative Stress Status

To evaluate the potential involvement of oxidative stress in GTFX-induced hepatotoxicity, intracellular ROS levels were measured after 48 h of exposure. Under this experimental condition, no significant differences in ROS fluorescence intensity were detected between GTFX-exposed larvae and the control group, despite pronounced morphological and histopathological alterations in the liver. These findings indicate that acute GTFX exposure did not lead to a detectable increase in intracellular ROS levels within the examined concentration range and time frame. Although oxidative stress is a well-recognized contributor to xenobiotic-induced liver injury, the absence of ROS elevation in the present study suggests that it may not be the dominant mechanism [38]. Similar patterns—structural liver injury without overt oxidative stress—have been reported in recent mechanistic studies of drug-induced liver injury, pointing to the involvement of alternative regulatory and metabolic pathways [39]. Accordingly, the present results point toward the contribution of non-oxidative processes to GTFX-induced hepatotoxicity.

### 4.4. Molecular Mechanism

To further explore the molecular basis of GTFX-induced hepatotoxicity, transcriptional changes in genes involved in lipid metabolism and xenobiotic biotransformation were examined. Acute GTFX exposure significantly upregulated *pparg*, *cyp1a*, and *cyp1b1* at the mRNA level, suggesting engagement of metabolic regulatory pathways in the zebrafish liver.

*pparg* encodes a nuclear receptor central to lipid metabolism, energy homeostasis, and adipogenic signaling in the liver [40]. Although hepatic expression of PPARγ is lower than in adipose tissue, its activation is associated with lipid accumulation and metabolic imbalance in the liver [41]. In the present study, increased *pparg* transcription is consistent with altered lipid metabolic regulation under GTFX exposure and with the observed phenotypic features, including increased liver opacity and delayed yolk sac utilization. Similar associations between PPARγ activation and lipid-associated liver injury have been reported in zebrafish and other vertebrate models [42].

In parallel, elevated transcription of *cyp1a* and *cyp1b1* reflects an increased demand for xenobiotic biotransformation. Members of the CYP1 family are key components of the hepatic detoxification system and are commonly induced as part of an adaptive response to chemical exposure, as documented in zebrafish and other experimental models. Importantly, induction of CYP1 enzymes is not necessarily accompanied by a detectable increase in ROS at a given time point, given the multiple factors that regulate cytochrome P450–linked ROS generation [43].

The transcription of *pparg*, *cyp1a*, and *cyp1b1* was coordinately upregulated, and no detectable ROS elevation was observed. These findings are consistent with a metabolism-associated hepatotoxicity pathway. These transcriptional changes are associative rather than causal. Collectively, these findings suggest that GTFX imposes metabolic stress on hepatocytes, leading to nuclear receptor-associated lipid dysregulation and increased xenobiotic processing demand, which may collectively contribute to the observed liver injury phenotype.

### 4.5. Relevance and Limitations of the Selected Concentration Range

The GTFX concentrations used in this study (0.2–2.3 mg/mL) are 2–3 orders of magnitude higher than environmentally detected levels (ng/L–µg/L). This disparity is intentional: the study was designed as an acute, high-dose toxicological investigation to maximize detection of mechanistic signals rather than to mimic environmental exposure. This concentration range is consistent with published acute toxicity data for other fluoroquinolones in zebrafish [23].

Regarding the concern that high concentrations may cause non-specific cytotoxicity, three lines of evidence support a pathway-specific interpretation. First, the observed changes are liver-specific (reduced area, increased opacity, delayed yolk absorption) rather than systemic. Second, gene expression changes are selective (upregulation of *pparg*, *cyp1a*, *cyp1b1*) without global transcriptional arrest. Third, mortality remained low (≤10%) even at the highest concentration, suggesting no extensive cytotoxicity. Nevertheless, we acknowledge that non-specific effects cannot be completely excluded.

These findings cannot be directly extrapolated to environmental risk assessment; rather, they provide a hypothesis-generating basis for future chronic, low-dose studies.

## 5. Conclusions

In this study, the hepatotoxic effects of GTFX were systematically evaluated in zebrafish larvae using integrated phenotypic, histopathological, and molecular analyses. Acute GTFX exposure caused pronounced alterations in liver development, including delayed yolk sac utilization, increased liver opacity, and reduced liver area, indicating impaired hepatic growth and metabolic stress during early development.

Histopathological analysis revealed hepatocyte swelling and cytoplasmic vacuolization, providing tissue-level evidence of structural liver injury. Notably, these morphological and histological changes occurred with no statistically significant difference in bulk ROS levels under the conditions tested.

At the molecular level, coordinated transcriptional upregulation of *pparg* and the CYP1 family genes (*cyp1a* and *cyp1b1*) was observed, indicating disrupted lipid metabolic regulation and an increased demand for xenobiotic biotransformation. Collectively, these findings are consistent with engagement of lipid metabolic and xenobiotic biotransformation pathways. Functional validation is needed in future studies.

The exposure concentrations used in this study exceed typical environmental levels. However, they were chosen to establish a clear mechanistic framework for interpreting fluoroquinolone-induced hepatotoxicity. The present results provide a foundation for future investigations focusing on chronic and low-dose exposures, which are needed to better assess the ecological and health risks associated with pharmaceutical contamination in aquatic environments.

## Figures and Tables

**Table 1:** Sequences of primers used for real-time quantitative PCR.

Target Gene	Primer Type	Primer Sequence (5′–3′)
*actb* (*β-actin*)	Forward	5′-TCGAGCAGGAGATGGGAACC-3′
	Reverse	5′-CTCGTGGATACCGCAAGATTC-3′
*pparg*	Forward	5′-CACTCGCTGGACATCAAGCC-3′
	Reverse	5′-TCCTGTAGCTGTACATGTGCGT-3′
*cyp1a*	Forward	5′-CGCTTGTATGGGCTTGTCCT-3′
	Reverse	5′-CGCAGCTAAAACAGGCACTC-3′
*cyp1b1*	Forward	5′-CCACCCGAACTCTGAAACTC-3′
	Reverse	5′-GTTTTCTTAGCCGCCTTCATTT-3′

## Data Availability

The original contributions presented in this study are included in the article. Further inquiries can be directed to the corresponding author(s).

## Abbreviations

The following abbreviations are used in this manuscript:

FQs	Fluoroquinolone antibiotics
GTFX	Gatifloxacin
3 dpf	3 days post-fertilization
MNLC	The maximum non-lethal concentration
LC_10_	10% lethal concentration
DCFH	2′,7′-dichlorodihydrofluorescein
DCF	2′,7′-dichlorofluorescein
A.U.	Arbitrary Units
ROS	reactive oxygen species
qPCR	quantitative real-time PCR
